# Work functioning impairment in the course of pharmacotherapy treatment for depression

**DOI:** 10.1038/s41598-020-72677-1

**Published:** 2020-09-24

**Authors:** Tomohisa Nagata, Yoshihisa Fujino, Makoto Ohtani, Kenji Fujimoto, Masako Nagata, Shigeyuki Kajiki, Makoto Okawara, Koji Mori

**Affiliations:** 1 Department of Occupational Health Practice and Management, Institute of Industrial Ecological Sciences, University of Occupational and Environmental Health, Japan, 1-1 Iseigaoka, Yahatanishi-ku, Kitakyushu, 807-8555 Japan; 2 Department of Environmental Epidemiology, Institute of Industrial Ecological Sciences, University of Occupational and Environmental Health, Japan, Kitakyushu, Japan; 3 Department of Information Systems Center, University of Occupational and Environmental Health, Japan, Kitakyushu, Japan; 4 Data Science Center for Occupational Health, University of Occupational and Environmental Health, Japan, Kitakyushu, Japan

**Keywords:** Health services, Occupational health

## Abstract

This study investigated the association between the duration of pharmacotherapy treatment for depression, or discontinuation from treatment, and work functioning impairment. This was a retrospective cohort study examining 30,409 workers. Work functioning impairment was assessed using a questionnaire, and treatment status was assessed using medical claims data. Odds ratios (ORs) of workers with severe work functioning impairment compared with healthy workers (control group) were calculated using logistic regression analysis. Continuous medical treatment was associated with severely impaired work functioning regardless of treatment period [continuous medical treatment; 4 months <: OR = 3.2, 4 months ≥, 10 months <: OR = 2.6, 10 months ≥, 14 months <: OR = 2.3, 14 months ≥, 16 months <: OR = 2.3, which are all statistically significant (p < 0.05)]. Workers who initially received pharmacotherapy treatment but discontinued in < 11 months had a significantly higher OR (treatment discontinuation period; 3 months <: OR = 2.3, 3 months ≥, 8 months <; OR = 2.0, 8 months ≥, 11 months <; OR = 3.0), while those who discontinued at ≥ 11 months did not (OR = 1.4, 95% CI 0.6–3.5). The sensitivity analysis excluding participants with at least one psychiatric comorbidity other than depression did not change the final result. It is important for the occupational health practitioners and attending psychiatrists to follow up in cooperation with each other, paying attention to the decrease in work functioning in addition to the symptoms.

## Introduction

Patients with depression are likely to experience many years lived with disability (YLDs). This is a global health issue because major depressive disorder (MDD) is among the five leading causes of YLDs worldwide, contributing 34.1 million of the total YLDs (805 million) as of 2016^1^. Depression reduces quality of life and affects work performance quality^2,3^. Presenteeism, defined as working while sick, is associated with reduced productivity and is a topic gaining increasing attention. A study found 6.4% of workers met the criteria for 12-month MDD, which is associated with 27.2 lost work days per sick worker per year and, in the United States, an overall loss of 225 million work days and $36.6 billion^4^. Based on total health-related costs due to absenteeism, presenteeism, and medical and pharmaceutical expenses, mental health disorders, including depression, comprise the largest burden of disease in the United States^5^ and Japan^6^. Our past study revealed that the presenteeism costs due to mental and behavioural disorders were $948 per 1000 full-time equivalent per year^6^. The other study revealed that sickness presence accounted for an average of 45.1 work days lost per employee per year^7^.

Undiagnosed and untreated depression had caused enormous socio-economic losses such as a decrease in labor force and an increase in social security costs due to the severity and chronicity of depression^8^. It has been shown that mild depression should be an intervention target because it has a high risk of becoming severe^9,10^. It is important to detect and treat depression during its early stages. Proper treatment throughout the acute phase can relieve related symptoms, and achieve remission and a return to full functioning and quality of life^10–13^. It is also strongly recommended to continue pharmacotherapy after remission following successful acute phase treatment for preventing relapse^11,14^. American Psychiatric Association guidelines recommend treating patients with primary depression with the same dose of antidepressants as used in the acute phase, for 4–9 months or longer after remission, and assuming good and consistent control of depression symptoms^11,15^. Meanwhile, discontinuation and dose reduction of antidepressant drugs in the early phases can increase the risk of exacerbating symptoms; early discontinuation of antidepressants results in a 77% increase in the risk of relapse and recurrence of depression in two years ^16^. In order to decide the treatment policy, it is important not only to monitor the patient’s psychiatric status but also to evaluate functional impairment and quality of life^11^.

Many depressed patients present with cognitive dysfunction such as slowed thoughts, poor concentration, distractibility, and reduce capacity to process information^11^. Understanding the type and extent of cognitive dysfunction is important in assessing the course of treatment. Attending physicians can evaluate functional impairment for their patients, and neuropsychological testing can be performed in the hospital^17^. However, it is difficult for the attending physician to intervene if the treatment is interrupted. If the patients were workers, the occupational health staff such as occupational physician and occupational health nurse can intervene with them in the workplace. Workers with depression tend to appear disabilities as work functions. Therefore, if the work functioning can be evaluated, it will be possible to find the timing of intervention by the occupational health staff. We previously developed and validated a self-administered questionnaire using the work functioning impairment scale (WFun) for assessment^18^. We also verified that the results of WFun assessments were correlated with the results of fit-to-work assessments conducted by occupational health nurses^19^.

Clinical treatment aims not only to relieve related symptoms, but to help patients recover their social functioning. Work functioning is an important social function for workers with depression. Information on the degree of work functioning in each phase of the treatment process is useful when deciding the necessity of cooperation between the occupational health staff and the attending physician, and when the attending physician decides the treatment content. However, to the best of our knowledge, there are no reports on changes in work functioning impairment based on treatment duration and discontinuation.

The purpose of this study is to describe the degree of work functioning impairment according to the course of pharmacotherapy treatment for depression (Objective 1: the duration of pharmacotherapy treatment, and Objective 2: the duration of discontinuation from pharmacotherapy treatment).

## Methods

This was a retrospective cohort study of 45,404 workers, from 13 companies in Japan. All 13 companies were manufacturers (five pharmaceutical, six automobile-related, one nonferrous metals, and one precision equipment).

### Data collection and ethical approval

The participants completed the WFun questionnaire between July and October 2015. The objectives of the study were explained to the participants, who were informed that participation was voluntary and that only the researchers, who were unaffiliated with the companies, would have access to the data. Ultimately, of the 45,404 workers, 33,415 (73.6%) participated.

We obtained participants’ medical claims data from the companies’ health insurance unions retrospectively, back to 15 months before the WFun questionnaire was given. These claims contained information on the date of any visits to a medical institution (outpatient or inpatient), the name of the institution, the disease(s), treatment/medication(s), and medical expenditures. Because Japan has a universal health insurance system, these claims data contained complete records on services used by the participants and covered under the system.

All methods were carried out in accordance with relevant guidelines and regulations. All data were labelled with unique codes assigned to each participant for personal information protection. Informed consent including implied consent was obtained from all subjects. The study was approved by the Ethics Committee of University of Occupational and Environmental Health, Japan (H26-026).

### Measurements for treatment of depression with pharmacotherapy

Medical claims data were used for determining whether the participants had visited medical institutions and received pharmacotherapy treatment for depression there each month within the 15 months prior to their completing the WFun questionnaire. Participants were determined to have received pharmacotherapy treatment if they satisfied both of the following two inclusion criteria and one exclusion criteria:

We included by two criteria as follow.

1. International Classification of Diseases 10 (ICD 10) codes F30–F39 (mood [affective] disorders) was given as the disease name.
2. Antidepressants and/or psychoneurotic drugs (therapeutic category of drugs in Japan: 117) were prescribed^20^.

We excluded workers who had International Classification of Diseases 10 (ICD 10) codes F30 (Manic episode) or F31 (Bipolar affective disorder) given as the disease name of the claims data. A total of 126 participants had F30 (Manic episode) or F31 (Bipolar affective disorder), and we excluded their data in the final analysis.

**Figure 1:**
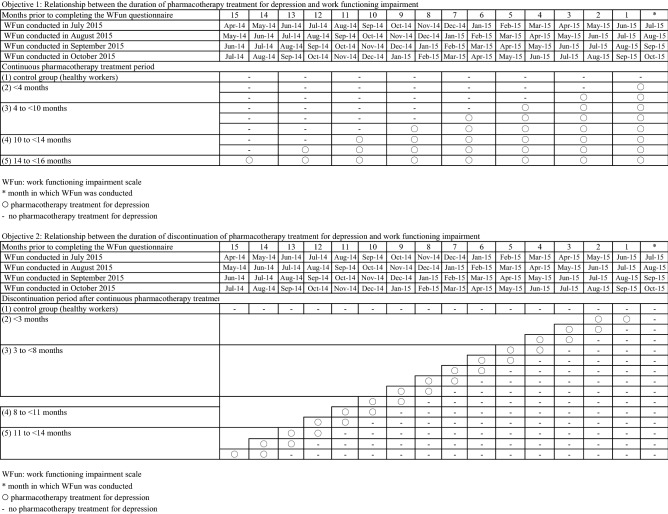
Definitions of the five categories of the study objectives.

Based on information obtained from medical claims data and WFun, the participants were divided into five categories in accordance with their duration of pharmacotherapy treatment (Objective 1) and duration of discontinuation from treatment (Objective 2). Detailed definitions are provided below and in Fig. 1.

Objective 1: Association between the duration of pharmacotherapy treatment for depression and work functioning impairment.

1. control group (healthy workers with no pharmacotherapy treatment): those who had received no related treatment within 15 months preceding the WFun
2. continuous pharmacotherapy treatment (< 4 months)
3. continuous pharmacotherapy treatment (4 to < 10 months)
4. continuous pharmacotherapy treatment (10 to < 14 months)
5. continuous pharmacotherapy treatment (14 to < 16 months).

A total of 315 participants were not classified under any of the above categories because they had received irregular pharmacotherapy treatment. Accordingly, we excluded their data in the final analysis.

Objective 2: Association between the duration of discontinuation of pharmacotherapy treatment for depression and work functioning impairment.

1. control group (healthy workers with no pharmacotherapy treatment): those who had received no related treatment within 15 months preceding the WFun
2. treatment discontinuation period (< 3 months)
3. treatment discontinuation period (3 to < 8 months)
4. treatment discontinuation period (8 to < 11 months)
5. treatment discontinuation period (11 to < 14 months).

A total of 546 participants were not classified under any of the above categories because they had received irregular pharmacotherapy treatment. Accordingly, we excluded their data in the final analysis.

### Outcome measurements

WFun is a self-administered questionnaire developed using the Rasch model^18^. It assesses the severity of work functioning impairment and has been validated in accordance with the Consensus-based Standards for the Selection of Health Measurement Instruments (COSMIN). Items of WFun and the scoring method was shown in Table 1. A higher total suggests greater work functioning impairment due to health problems. A total of ≥ 21 points suggests severely impaired work functioning: a previous study found approximately 20% of employees had a WFun score of 21 points, and an assessment by occupational health nurses found these people were likely to have severely impaired work functioning and that they demonstrated high detectability with an area under the ROC curve of 0.83^19^. Cronbach’s alpha coefficient for the seven items of WFun was 0.92 in this study.

**Table 1:** Items of Work Functioning Questionnaire (WFun) and the scoring method.

	Options and score of each option				
	Not at all	≥ 1 days a month	About 1 day a week	≥ 2 days a week	Almost every day
**Items of Work Functioning Questionnaire (WFun)**					
(1) I have not been able to behave socially	1	2	3	4	5
(2) I have not been able to maintain the quality of my work	1	2	3	4	5
(3) I have had trouble thinking clearly	1	2	3	4	5
(4) I have taken more rests during my work	1	2	3	4	5
(5) I have felt that my work is not going well	1	2	3	4	5
(6) I have not been able to make rational decisions	1	2	3	4	5
(7) I have not been proactive about my work	1	2	3	4	5

The total score is the sum of the scores of all items. The score range is 7 to 35 points. A high score means that the work impairment is large.

### Other measurements

We used a self-administered questionnaire to determine the participants’ sex, age, job type and job title. Age in years was categorised into five groups (< 30, 30–39, 40–49, 50–59, and ≥ 60 years), as was job type (clerical and administrative support, sales, research and development, production line, and other), while job title was categorised as manager or rank-and-file employee.

### Statistical analysis

In addressing each of the two objectives indicated above, we performed logistic regression analyses using each category as an explanatory variable and a total WFun score of ≥ 21 points as an outcome variable. Using the category of no pharmacotherapy treatment (healthy workers) as a reference, we calculated the OR (and 95% CI) for each category. Stata version 14.2 (StataCorp LLC; TX, USA) was used for analyses, with significance set at < 0.05.

### Sensitivity analysis

Many patients can be expected to have at least one psychiatric comorbidity and this can quite significantly alter work functioning. Sensitivity analysis was performed in order to confirm that the results did not change even if these individuals were excluded. We excluded participants below (exclusion criteria) and performed logistic regression analyses in the same way.

#### Exclusion criteria

1. International Classification of Diseases 10 (ICD 10) codes F00–F09 (organic, including symptomatic, mental disorders) was given as the disease name. F00-09 includes F00 (Dementia in Alzheimer disease) and F01 (Vascular dementia).
2. International Classification of Diseases 10 (ICD 10) codes F10–F19 (mental and behavioural disorders due to psychoactive substance use) was given as the disease name. F10–F19 includes F10 (mental and behavioural disorders due to use of alcohol) and F11 (mental and behavioural disorders due to use of opioids).
3. International Classification of Diseases 10 (ICD 10) codes F20–F29 (schizophrenia, schizotypal and delusional disorders).

## Results

### Participants’ characteristics

Among the 33,415 participants, we were unable to obtain the medical claims data of 2,533 participants and 473 did not answer at least one of the seven WFun questions. After excluding them, we had data from 30,409 participants for analysis.

Table 2 shows the participants’ characteristics. Male employees comprised 85% of the participants, and the largest proportion of participants (32%) were 40–49 years old, followed by 30–39 (24%), 50–59 (22%). Participants primarily worked in research and development (20%), clerical and administrative support (18%), and sales (18%).

**Table 2:** Participants' characteristics.

	N	%
**Sex**		
Men	25,882	85
Women	4527	15
**Age (years)**		
≤ 29	5681	19
30–39	7448	24
40–49	9786	32
50–59	6621	22
≥ 60	873	3
**Job type**		
Clerical and administrative support	5423	18
Sales	5390	18
Research and development	5939	20
Production line	2845	9
Other	10,569	35
Missing	243	1
**Job title**		
Manager	5394	18
Rank-and-file employee	15,555	51
Missing	9460	31

#### Objective 1

Table 3 shows the association between the duration of pharmacotherapy treatment for depression and work functioning impairment.

**Table 3:** Number of workers, mean WFun score, and proportion of workers with a high WFun score (21 points or more), and logistic regression analyses for Objective 1: association between the duration of medical treatment for depression and work functioning impairment.

	N	WFun score		WFun score ≥ 21	OR	95% CI		p value
		Mean	SD	%				
(1) Control group (healthy workers)	29,564	14.7	6.4	20	Reference			
(2) Continuous medical treatment (4 months <)	63	19.1	8.1	44	3.2	1.9	5.2	< 0.001
(3) Continuous medical treatment (4 months ≥, 10 months <)	58	18.7	8.1	40	2.6	1.5	4.4	< 0.001
(4) Continuous medical treatment (10 months ≥, 14 months <)	33	18.5	7.8	36	2.3	1.1	4.6	0.024
(5) Continuous medical treatment (14 months ≥ , 16 months <)	250	17.9	7.3	37	2.3	1.8	3.0	< 0.001

*OR* odds ratio, *CI* confidence interval.

Continuous medical treatment was associated with severely impaired work functioning regardless of treatment period [continuous medical treatment; 4 months <: OR = 3.2, 4 months ≥, 10 months <: OR = 2.6, 10 months ≥, 14 months <: OR = 2.3, 14 months ≥, 16 months <: OR = 2.3, which are all statistically significant (p < 0.05)].

#### Objective 2

Table 4 shows the association between the duration of discontinuation from pharmacotherapy treatment for depression and work functioning impairment.

**Table 4:** Number of workers, mean WFun score, and proportion of workers with a high WFun score (21 points or more), and logistic regression analyses for Objective 2: association between the duration of discontinuation from medical treatment for depression and work functioning impairment.

	N	WFun score		WFun score ≥ 21	OR	95% CI		p value
		Mean	SD	%				
(1) Control group (healthy workers)	29,564	14.7	6.4	20	Reference			
(2) Treatment discontinuation period (3 months <)	81	17.9	6.8	37	2.3	1.5	3.7	< 0.001
(3) Treatment discontinuation period (3 months ≥, 8 months <)	48	19.1	5.8	33	2.0	1.1	3.6	0.026
(4) Treatment discontinuation period (8 months ≥, 11 months <)	21	19.2	6.8	43	3.0	1.3	7.1	0.014
(5) Treatment discontinuation period (11 months ≥, 14 months <)	23	16.2	6.7	26	1.4	0.6	3.5	0.481

*OR* odds ratio, *CI* confidence interval.

Workers who initially received pharmacotherapy treatment but discontinued in < 11 months had a significantly higher OR (treatment discontinuation period; 3 months <: OR = 2.3, 3 months ≥, 8 months <; OR = 2.0, 8 months ≥, 11 months <; OR = 3.0), while those who discontinued at ≥ 11 months did not show a significantly higher risk of work functioning impairment (OR = 1.4, 95% CI 0.6–3.5).

A sensitivity analysis was performed on both objectives 1 and 2. The results excluding participants with at least one psychiatric comorbidity other than depression did not change (Supplementary Table S1, S2).

## Discussion

This study investigated the association between the duration of pharmacotherapy treatment and work functioning impairment as well as the association between the duration of discontinuation from pharmacotherapy treatment and work functioning impairment. Continuous medical treatment was associated with severely impaired work functioning regardless of treatment period. Further, workers who initially received pharmacotherapy treatment but discontinued in < 11 months had a significantly higher OR compared to healthy workers (control group), while those who discontinued at ≥ 11 months did not (OR = 1.4, 95% CI 0.6–3.5).

The present results have several implications for future interventions. First, in this study, the risk of experiencing severe work functioning impairment was higher even with ≥ 1 year of antidepressant treatment. While most recent systematic reviews have shown antidepressant treatment improves functional outcomes, one reported that 60% of patients had persistent functional impairment even 6 months after symptom remission^21^. Job performance is also improved over time following symptom amelioration^22^. These results are consistent with the present findings. Based on these findings, if the symptoms were recovered by the treatment of antidepressants but the work function was deteriorated, it may be necessary to consider a side effects of antidepressants or a cognitive impairment due to causes other than depression such as Alzheimer’s disease.

Second, in addition to symptom amelioration, job-related functioning and performance are important for continued employment. Our study suggests the importance of monitoring patients for ≥ 1 year after finishing treatment (Table 4), assuming work performance is only restored after recovery from symptoms. The risk of work functioning impairment is markedly higher, particularly in the acute phase (< 4 months as shown in Table 3), wherein the risk is threefold that of healthy controls. It is therefore important to assess workers’ degree of recovery from impairment after starting treatment, even if their symptoms have remitted. Additionally, given that severe work functioning impairment (WFun ≥ 21 points) can increase the risk of taking sick leave in the future, such assessments are important for preventing depression-related work leave^23^.

Third, in the present study, the risk of severe work functioning impairment remained high for about 1 year after discontinuation of antidepressant treatment (Table 4). This is the first study to assess work functioning following treatment discontinuation. To deter workers impatience with their recovery, attending psychiatrists or occupational physicians should explain the likelihood of being at an increased risk of work functioning impairment for ≥ 1 year after discontinuing antidepressant treatment. This explanation may give motivation to seek medical assistance when experiencing a symptom relapse. Additionally, Japanese companies with > 50 employees are legally obliged to employ corporate healthcare professionals, mainly occupational physicians, in the workplace. In these companies, importance should be placed on collaboration between the attending psychiatrist and healthcare professionals via the patient, and these healthcare professionals should follow-up patients after the patients discontinue antidepressant treatment.

The present study has a number of strengths. It is the first study to investigate work functioning impairment in employees across several phases of antidepressant treatment for depression by comparing findings with healthy workers who have never received such treatment. The study also examined work functioning impairment across several phases after discontinuation of pharmacotherapy treatment for depression. Additionally, this study used a large-scale, workplace cohort and was based on mutually exclusive and collectively exhaustive medical claims data obtained via Japan’s universal health insurance system.

The study does also have some limitations. First, we did not obtain information on the responsiveness of treatment for depression and the reason for discontinuation of treatment. We could not evaluate the effects of these situations on work functioning.

Second, we did not assess depression severity. We therefore did not account for its effect on work functioning impairment. However, a recent systematic review reported, based on several studies, that although antidepressant treatment ameliorates functional outcomes, the severity of depression at the start of treatment does not affect this amelioration^2^. We were also unable to examine non-pharmaceutical treatments, such as psychotherapy, concurrent with pharmacotherapy; therefore we did not evaluate the effects of such treatments. Additionally, we did not examine other aspects, such as the effects of job styles at certain workplaces, with relation to work performance. Finally, because the study population comprised employees from companies listed on the First Section of the Tokyo Stock Exchange, and thus generally representing those of higher socioeconomic status, care should be taken in generalising the findings. Furthermore, roughly half were pharmaceutical company employees and therefore likely have greater medical knowledge than the general public. People with higher socioeconomic attributes, such as higher education levels, are also more accepting of mental health services^24^. The proportion of participants who had depression but were not using such services was therefore likely lower than that in the general population. This limitation should be taken into account when interpreting the results. Those taking oral antidepressants accounted for 2.7% of all participants, which is similar to the proportion in a large-scale survey on mood (emotional) disorders among general residents in Japan (in which the 12-month prevalence of depression was 2.1%)^25^.

## Conclusions

The present study showed an association between the duration of pharmacotherapy treatment and work functioning impairment as well as the relationship between the duration of discontinuation from treatment and such impairment. Patients were found to be at an increased risk of severe work functioning impairment for ≥ 1 year after discontinuing antidepressants. It is important for the occupational health practitioners and attending psychiatrists to follow up in cooperation with each other, paying attention to the decrease in work functioning in addition to the symptoms.

## Supplementary information

Supplementary Tables.

## Data Availability

The analysis presents clinical data of a large-scale workplace-based cohort with ongoing follow-up examinations. This project constitutes a major scientific effort, therefore data are not made available for the scientific community outside. Interested researchers make their requests to the leader of the Collabo-Health Study (Tomohisa Nagata; tomohisa@med.uoeh-u.ac.jp).
