# Supplementary material for: Work functioning impairment in the course of pharmacotherapy treatment for depression
Source: Sci Rep. 2020 Sep 24;10:15712. doi: 10.1038/s41598-020-72677-1 (PMC7519139; doi:10.1038/s41598-020-72677-1)
Supplement: Supplementary file 1 — Supplementary Tables. [file 41598_2020_72677_MOESM1_ESM.docx]

Supplementary Information for:

**Work functioning impairment in the course of pharmacotherapy treatment for depression**

Tomohisa Nagata^1*^, Yoshihisa Fujino^2^, Makoto Ohtani^3^, Kenji Fujimoto^4^, Masako Nagata^1, 4^, Shigeyuki Kajiki^1^, Makoto Okawara^2^, Koji Mori^1^

1 Department of Occupational Health Practice and Management, Institute of Industrial Ecological Sciences, University of Occupational and Environmental Health, Japan

2 Department of Environmental Epidemiology, Institute of Industrial Ecological Sciences, University of Occupational and Environmental Health, Japan

3 Department of Information Systems Center, University of Occupational and Environmental Health, Japan

4 Data Science Center for Occupational Health, University of Occupational and Environmental Health, Japan

*Address correspondence to: Tomohisa Nagata, MD, PhD, Department of Occupational Health Practice and Management, Institute of Industrial Ecological Sciences, University of Occupational and Environmental Health, 1-1 Iseigaoka, Yahatanishi-ku, Kitakyushu 807-8555, Japan

E-mail: tomohisa@med.uoeh-u.ac.jp; Tel: +81-93-603-1611; Fax: +81-93-603-2155

Supplementary Table S1. Number of workers, mean WFun score, and proportion of workers with a high WFun score (21 points or more), and logistic regression analyses for Objective 1: Association between the duration of medical treatment for depression and work functioning impairment (results of sensitivity analysis excluding participants with at least one psychiatric comorbidity other than depression).

|  | N | WFun score | |  | WFun score ≥21 | OR | 95%CI | | p value |
| --- | --- | --- | --- | --- | --- | --- | --- | --- | --- |
|  |  | Mean | SD |  | % |  |  |  |  |
| (1) control group (healthy workers) | 29414 | 14.7 | 6.4 |  | 20 | reference |  |  |  |
| (2) continuous medical treatment (4 months<) | 60 | 19.0 | 8.0 |  | 43 | 3.0 | 1.8 | 5.1 | <0.001 |
| (3) continuous medical treatment (4 months≥, 10 months<) | 55 | 18.4 | 8.0 |  | 38 | 2.4 | 1.4 | 4.2 | 0.001 |
| (4) continuous medical treatment (10 months≥, 14 months<) | 31 | 18.0 | 7.6 |  | 32 | 1.9 | 0.9 | 4.0 | 0.098 |
| (5) continuous medical treatment (14 months≥, 16 months<) | 221 | 17.6 | 7.1 |  | 34 | 2.0 | 1.5 | 2.7 | <0.001 |
|  |  |  |  |  |  |  |  |  |  |
| OR: odds ratio | | | | | | | | | |
| CI: confidence interval | | | | | | | | | |

Supplementary Table S2. Number of workers, mean WFun score, and proportion of workers with a high WFun score (21 points or more), and logistic regression analyses for Objective 2: Association between the duration of discontinuation from medical treatment for depression and work functioning impairment (results of sensitivity analysis excluding participants with at least one psychiatric comorbidity other than depression).

|  | N | WFun score | |  | WFun score ≥21 | OR | 95%CI | | p value |
| --- | --- | --- | --- | --- | --- | --- | --- | --- | --- |
|  |  | Mean | SD |  | % |  |  |  |  |
| (1) control group (healthy workers) | 29414 | 14.7 | 6.4 |  | 20 | reference |  |  |  |
| (2) treatment discontinuation period (3 months<) | 75 | 17.7 | 6.7 |  | 37 | 2.4 | 1.5 | 3.8 | <0.001 |
| (3) treatment discontinuation period (3 months≥, 8 months<) | 46 | 18.9 | 5.9 |  | 33 | 1.9 | 1.0 | 3.6 | 0.039 |
| (4) treatment discontinuation period (8 months≥, 11 months<) | 20 | 18.8 | 6.7 |  | 40 | 2.6 | 1.1 | 6.5 | 0.033 |
| (5) treatment discontinuation period (11 months≥, 14 months<) | 22 | 15.8 | 6.6 |  | 23 | 1.2 | 0.4 | 3.2 | 0.763 |
|  |  |  |  |  |  |  |  |  |  |
| OR: odds ratio | | | | | | | | | |
| CI: confidence interval | | | | | | | | | |
